# Heterologous Expression and Maturation of an NADP-Dependent [NiFe]-Hydrogenase: A Key Enzyme in Biofuel Production

**DOI:** 10.1371/journal.pone.0010526

**Published:** 2010-05-06

**Authors:** Junsong Sun, Robert C. Hopkins, Francis E. Jenney, Patrick M. McTernan, Michael W. W. Adams

**Affiliations:** Department of Biochemistry and Molecular Biology, University of Georgia, Athens, Georgia, United States of America; University Paris Diderot-Paris 7, France

## Abstract

Hydrogen gas is a major biofuel and is metabolized by a wide range of microorganisms. Microbial hydrogen production is catalyzed by hydrogenase, an extremely complex, air-sensitive enzyme that utilizes a binuclear nickel-iron [NiFe] catalytic site. Production and engineering of recombinant [NiFe]-hydrogenases in a genetically-tractable organism, as with metalloprotein complexes in general, has met with limited success due to the elaborate maturation process that is required, primarily in the absence of oxygen, to assemble the catalytic center and functional enzyme. We report here the successful production in *Escherichia coli* of the recombinant form of a cytoplasmic, NADP-dependent hydrogenase from *Pyrococcus furiosus,* an anaerobic hyperthermophile. This was achieved using novel expression vectors for the co-expression of thirteen *P. furiosus* genes (four structural genes encoding the hydrogenase and nine encoding maturation proteins). Remarkably, the native *E. coli* maturation machinery will also generate a functional hydrogenase when provided with only the genes encoding the hydrogenase subunits and a single protease from *P. furiosus*. Another novel feature is that their expression was induced by anaerobic conditions, whereby *E. coli* was grown aerobically and production of recombinant hydrogenase was achieved by simply changing the gas feed from air to an inert gas (N_2_). The recombinant enzyme was purified and shown to be functionally similar to the native enzyme purified from *P. furiosus*. The methodology to generate this key hydrogen-producing enzyme has dramatic implications for the production of hydrogen and NADPH as vehicles for energy storage and transport, for engineering hydrogenase to optimize production and catalysis, as well as for the general production of complex, oxygen-sensitive metalloproteins.

## Introduction

From the human economic perspective, molecular hydrogen (H_2_) is an ideal form of transportable energy. It is superior to current forms such as gasoline as it is less toxic, safer to use, and has an approximately three-fold higher energy yield per kilogram [1]. A number of problems must be surmounted before a ‘hydrogen economy’ is a reality, however, including the economic production and transportation of H_2_ [2], [3]. At present H_2_ is generated from fossil fuels by steam reforming processes and by electrolysis of water using electricity typically produced from fossil fuels [1]–[3]. Biological H_2_ production is an attractive, and renewable, alternative for human needs. The gas is generated by microorganisms from all three domains of life, and for some, H_2_ provides an excellent source of low potential reducing power (H_2_/H^+^, E_o_' = −420mV) for growth and biosynthesis [4], [5]. Microorganisms have evolved elegant and efficient mechanisms for H_2_ metabolism. All are based on the enzyme hydrogenase, which catalyzes the reversible reduction of protons (Eqn. 1):
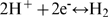
(1)


Many microorganisms obtain energy by metabolically coupling H_2_ oxidation to a variety of terminal electron acceptors such as fumarate, sulfate, carbon dioxide, and oxygen [4], [6]. The production of H_2_ is typical of organisms occupying anaerobic environments and is used as a means of disposing of excess reductant from oxidative metabolism.

Hydrogenases catalyze H_2_ production using a binuclear metal center active site consisting of either [NiFe] or [FeFe] [6], [7]. The [FeFe]-hydrogenases usually have high catalytic rates but are extremely sensitive to irreversible inactivation by oxygen, much more so than the [NiFe]-enzymes [4]. In contrast to [FeFe]-hydrogenases, which are limited to certain bacteria and a few unicellular eukaryotes, [NiFe]-hydrogenases are widespread in both bacteria and archaea. Although these two classes of hydrogenase are not phylogenetically related, the geometry of their catalytic sites is highly similar [7], and both contain the unusual biological ligands carbon monoxide (CO) and cyanide (CN) which serve to modulate their electronic structure and facilitate catalysis [8].

**Figure 1:**
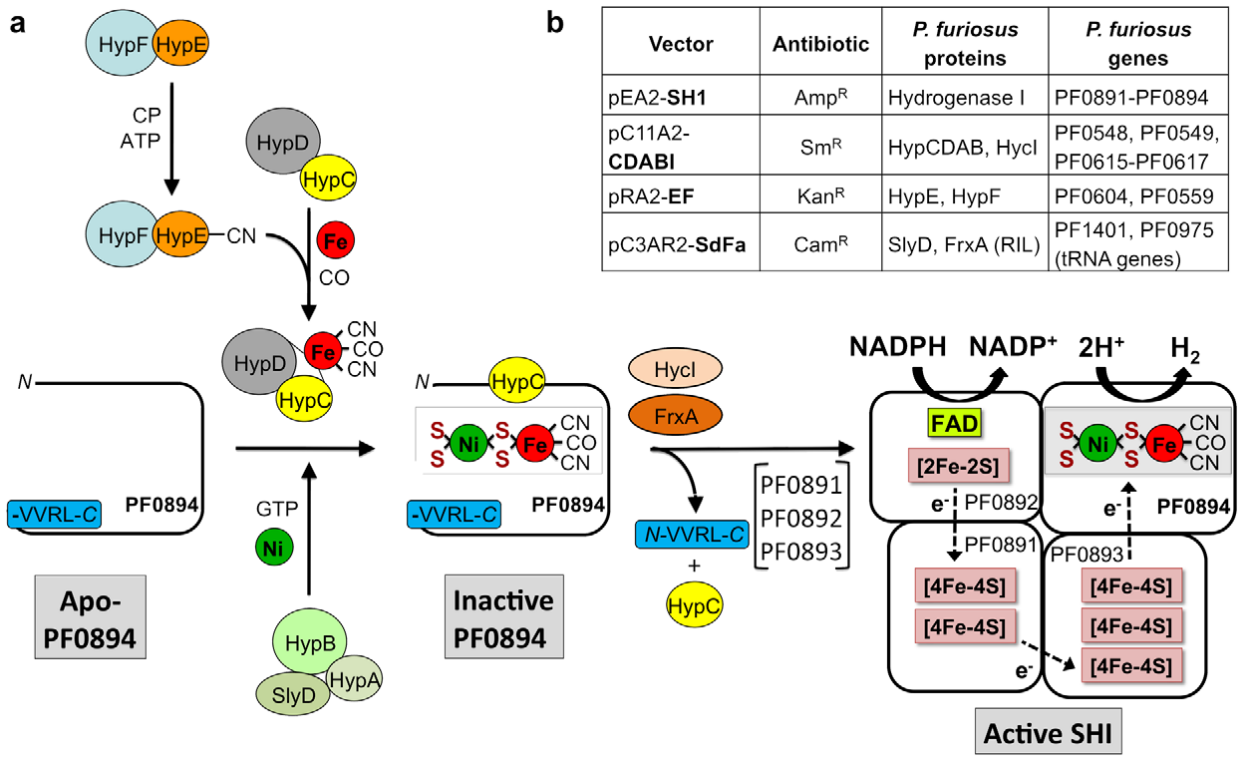
Synthesis of *P. furiosus* soluble hydrogenase I (SHI) and the plasmid constructs used for recombinant production. (**a**) Maturation pathway for the [NiFe] catalytic site of *P. furiosus* SHI is based on that proposed for *E. coli* hydrogenase 3 [10]. HypE and HypF generate thiocyanate in an ATP-dependent process from carbamoyl phosphate (CP). The source of the CO ligand is unknown. The HypCD complex binds the iron atom containing the CN and CO ligands and donates it to the catalytic subunit (PF0894). The Ni atom is provided by the HypAB-SlyD complex in a GTP-dependent reaction. The Ni and Fe atoms in the [NiFe] site are coordinated to PF0894 by the S atoms of four of its Cys residues (indicated by S). Activation of PF0894 requires cleavage of four C-terminal residues (-VVRL) catalyzed by a newly identified peptidase, FrxA (and to a much lesser extent by HycI). It is not known how the biosynthesis of PF0894 is coordinated with that of the other three subunits (PF0891, PF0892 and PF0893) to give catalytically-active SHI. The predicted cofactor content of mature heterotetrameric SHI [11] is shown together with their roles in the reversible production of hydrogen gas from NADPH. The abbreviations used are: CP, carbamoyl phosphate; CO, carbonyl ligand; CN, cyano ligand; FAD, flavin adenine dinucleotide. (**b**) The expression plasmids utilized for production of SHI in *E. coli* strain MW4W. The antibiotics refer to the selective marker on each plasmid: Amp, Ampicillin; Sm, Streptomycin; Kan, Kanamycin; Cam, Chloramphenicol.

All [NiFe]-hydrogenases are comprised of at least two subunits, one of which (the large subunit, or LSU) contains the [NiFe] catalytic site, while the other (the small subunit, or SSU) contains three iron-sulfur (FeS) clusters. These clusters transfer electrons from an external electron donor to the [NiFe] active site in the LSU for reduction of protons. The assembly of the [NiFe] site and maturation of the enzyme requires the concerted reactions of multiple gene products. The facultative anaerobe *E. coli* contains three membrane-bound hydrogenases, and elegant studies by Böck and coworkers have shown that the assembly of one of them (HYD3) requires the participation of eight accessory proteins (encoded by *hypA-hypF, hycI*, and *slyD*, Fig. 1a) [9]. The final step of hydrogenase maturation requires a specific protease (HycI) that removes a peptide of ≤20 residues from the C-terminal of the LSU, allowing the protein to fold around the [NiFe] active site [12]. In contrast, it appears that only three maturation proteins are required for the production of a functional [FeFe]-hydrogenase [13].

In the present study we demonstrate for the first time the heterologous expression in a genetically-tractable organism (*E. coli*) of a functional [NiFe]-hydrogenase enzyme, that from a model hyperthermophilic microorganism, *Pyrococcus furiosus* (*Pf*), which grows optimally at 100°C [14]. Previous attempts to heterologously produce [NiFe]-hydrogenases have involved related host organisms and/or have met with limited success. For example, *Desulfovibrio gigas* hydrogenase produced in *D. fructosovorans* exhibited only low activity [15], and transfer of the entire H_2_ uptake (*hup*) gene cluster (∼18 kb) from *Rhizobium leguminosarum* led to functional expression in only some closely-related species [16]. A functional, NAD-dependent [NiFe] hydrogenase from a gram-positive organism, *Rhodococcus opacus,* was produced in the gram-negative *Ralstonia eutropha* [17], and the membrane bound hydrogenase of *Ralstonia eutropha* was produced in *Pseudomonas stutzeri* using a broad-host-range plasmid containing the *hyp* genes and a regulatory hydrogenase-histidine kinase system [18]. This H_2_-sensing, regulatory hydrogenase of *R. eutropha* has also been heterologously produced in *E. coli* but this does not require the proteolytic processing involved in maturation of the [NiFe]-hydrogenase enzymes [19]. While previous attempts to heterologously produce a recombinant [NiFe]-hydrogenase in *E. coli* have been unsuccessful [20], [21], our system employs a novel set of compatible vectors modified with an anaerobically-induced *E. coli* hydrogenase promoter, P_hya_, and minimally requires a single processing gene from *Pf*. It therefore appears that the *E. coli* processing enzymes can assemble functional (and soluble) [NiFe]-hydrogenase enzyme from phylogenetically distant organisms, even from a completely different domain of life, including those growing at extreme temperatures.

*Pf* grows by fermenting carbohydrates to organic acids, CO_2_ and H_2_ [14]. It contains three [NiFe]-hydrogenases; two are cytoplasmic enzymes consisting of four subunits, and the third is a membrane-bound hydrogenase (MBH) of fourteen subunits. The two cytoplasmic enzymes, termed soluble hydrogenase I (SHI) and hydrogenase II (SHII), utilize NADP(H) as the physiological electron carrier [22]. MBH accepts electrons from reduced ferredoxin (Fd_red_) and is the enzyme responsible for evolving hydrogen during growth, while SHI and SHII are thought to be involved in the recycling of hydrogen produced by MBH [23]. Herein we demonstrate heterologous production of functional *Pf*SHI.

## Results

*Pf* SHI is encoded by four genes (PF0891-0894) in a single operon, with the catalytic LSU encoded by PF0894 and SSU encoded by PF0893 [22]. One of the additional subunits is predicted to contain two FeS clusters (PF0891) while the other (PF0892) is predicted to contain another FeS cluster and flavin adenine dinucleotide (FAD), and to interact with NAD(P)(H). SHI purified from *P. furiosus* biomass is extremely stable at high temperatures (t_1/2_ for loss of activity ∼2 hours at 100°C) and in the presence of oxygen (t_1/2_ ∼6 hours at 23°C in air) [11].

The four structural genes encoding *Pf* SHI (PF0891-PF0894) were coexpressed in *E. coli* under anaerobic conditions with eight processing genes identified in the *Pf* genome (*hypCDABEF, hycI* and *slyD*), based on sequence similarity to the processing genes identified in *E. coli*. These include PF0617, which is the homolog of the *E. coli* peptidase (HycI) involved in C-terminal processing of one of its three hydrogenases, termed HYD3. However, the C-terminal region of *Pf* SHI is more similar to the catalytic subunit of another *E. coli* hydrogenase, HYD2, rather than to HYD3, and HYD2 is processed by another protease (HybD) [24]. A search of the *Pf* genome revealed a gene (PF0975), annotated as ferredoxin oxidoreductase A (*frxA*), which shows 47% sequence similarity to *E. coli* HybD. This was included as the ninth *Pf* gene to be coexpressed in *E. coli* under anaerobic conditions.

**Figure 2:**
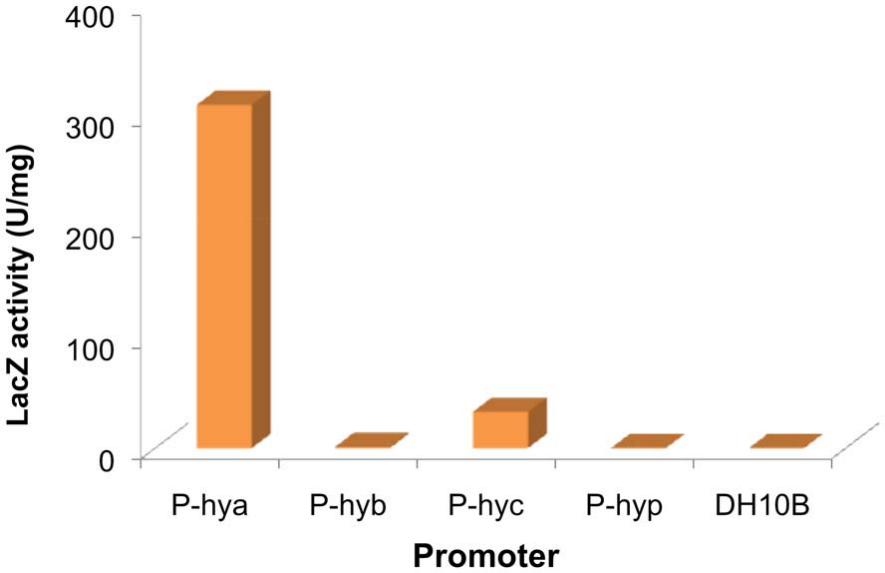
Analysis of *E. coli* promoters with a *lacZ* gene fragment β-galactosidase reporter assay. Plasmids were transformed into DH10B. The specific activity of *lacZ* was measured by a modified Miller [30] assay calculated as 200(OD_420-t1_-OD_420-t2_) min^−1^mg^−1^, where t1 and t2 are the start and end time points, respectively.

**Figure 3:**
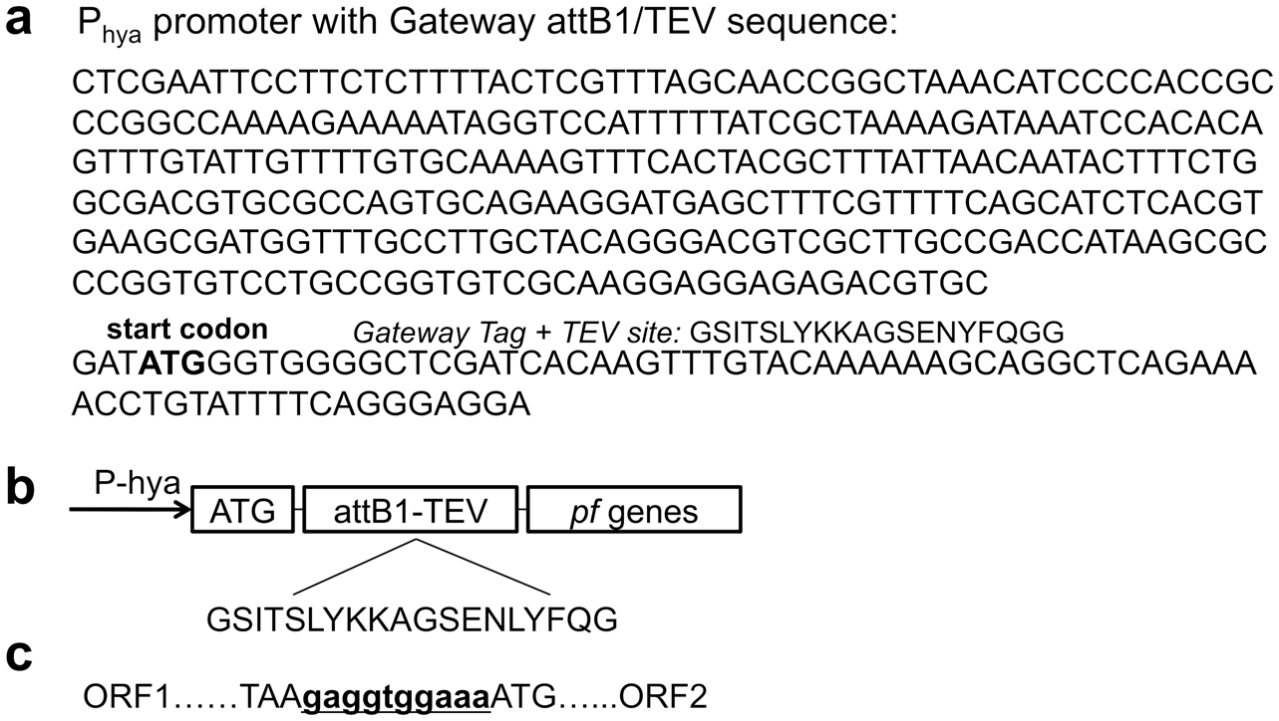
Plasmid construction for rSHI production in *E. coli*. P_hya_ was cloned into a set of patented plasmids constructed by inserting Invitrogen Gateway cassettes into Novagen Duet plasmids [25]. (**a**) Sequences of *E. coli* anaerobic promoter P_hya_, attB1 and TEV protease recognition site sequence and their encoded peptide sequence [31]. (**b**) Elements constructed in the final expression plasmids: P_hya_ promoter, N-terminus of the first protein in the operon derived from the Gateway recombination cloning site (attB1) including a TEV protease site [31] and *P. furiosus* genes. (**c**) Artificial Shine-Dalgarno sequences inserted between *P. furiosus* fused ORFs in plasmids pC11A2-CDABI, pC3AR2-SdFa and pRA2-EF.

A series of four compatible vectors based on the DUET vector system (Novagen) had been modified previously to contain GATEWAY™ recombination sites (Invitrogen) [25], which allows coexpression of as many as eight separate genes or operons under the control of T7 promoters. This cloning approach also inserts 19 amino acids (GSITSLYKKAGSENYFQGG, ∼2.0 kDa) at the N-terminus of the first gene of the operon in each plasmid. Since the standard T7 promoter does not function in *E. coli* under anaerobic conditions, four different promoters were investigated for anaerobic heterologous expression of the *Pf* genes in *E. coli*. Three were native *E. coli* hydrogenase promoters that are anaerobically-induced (*hya, hyb,* and *hy*c, [26]-[28]), while the fourth (*hyp*, [29]) induces expression of *E. coli* hydrogenase-processing genes. Expression of the *lacZ* gene by these four promoters (Fig. 2) and several of the *Pf hyd* genes, including *hypCDAB* and *hycI*, were examined using RT-PCR (data not shown). The *hya* promoter (P_hya_) was induced by anaerobiosis and gave the highest level of expression. The T7 promoters on all expression vectors were therefore replaced with P_hya_ (Fig. 3). One vector (pDEST-C3A) was further modified to include the tRNA genes from plasmid pRIL (Stratagene), which is typically required for efficient expression of *Pf* genes in *E. coli* due to differing codon usage, creating the plasmid pC3A-RIL. The complete list of expression vectors used in this study is given in Table 1.

**Table 1:** Strains and plasmids used in this study.

Strains and plasmids	Genotype	Source
**Strains**		
*E. coli* MW1001	*lacI* ^q^ *rrnB*_T14_ *ΔlacZ*_WJ16_ *hsdR514 ΔaraBAD*_AH33_ *ΔrhaBAD*_LD78_ *ΔhyaB ΔhybCΔhycE*	32
*E. coli* DH10B	*F^−^ endA1 recA1 galE15 galK16 nupG rpsL ΔlacX74 Φ80lacZΔM15 araD139 Δ(ara,leu)7697 mcrA Δ(mrr-hsdRMS-mcrBC) λ^−^*	Invitrogen
*E. coli* MW4W	MW1001 strain with plasmids: pC11A2-CDABI/pC3AR2-SF/pRA2-EF/pEA2-SH1	This study
**Plasmids**		
**a. lacZ reporter plasmids**		
pSB2019	Amp^R^ P_xylA_-GFP	33
pLXA	Amp^R^ P_hya_-lacZ	This study
pLXB	Amp^R^ P_hyb_-lacZ	This study
pLXC	Amp^R^ P_hyc_-lacZ	This study
pLXP	Amp^R^ P_hyp_-lacZ	This study
**b. Destination and Expression plasmids**		
pDEST-C1	Cloning vector, Sm^R^	25
pDEST-C11A2	Cloning vector, Sm^R^	This study
pC11A2-CDABI	Expression vector, Sm^R^	This study
pDEST-C3	Cloning vector, Cm^R^	25
pDEST-C3A	Cloning vector, Cm^R^	This study
pRIL	Express tRNA argU/ileY/leuW, Cm^R^	Stratagene
pC3A-RIL*	Cloning vector, Cm^R^	This study
pC3AR2-slyD*	Expression vector, Cm^R^	This study
pC3AR2-SdFa*	Expression vector, Cm^R^	This study
pET-CAG2	Cloning vector, Amp^R^	This study
pEA2-SH1	Expression vector, Amp^R^	This study
pET-A-SH1	Expression vector, Amp^R^	This study
pRSFDuet-1	Cloning vector, Kan^R^	Novagen
pRSF-CAG2	Cloning vector, Kan^R^	This study
pRA2-EF	Expression vector, Kan^R^	This study

*Contain tRNA genes *argU/ileY/leuW*.

**Figure 4:**
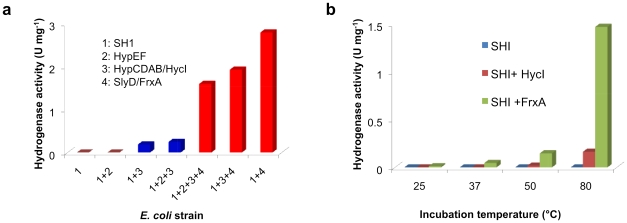
Accessory proteins required for maturation of recombinant *P. furiosus* SHI *in vivo* and *in vitro*. (**a**) Recombinant SHI (rSHI) was expressed in the presence of different *Pf* processing genes and the resulting *E. coli* cell-extracts were assayed at 80°C. The bars indicate methyl viologen-linked specific activity expressed as U mg^−1^ or µmol H_2_ evolved min^−1^ mg^−1^. FrxA but not SlyD is necessary to produce high amounts of active hydrogenase. (**b**) Processing of rSHI in cell-free extracts of *E. coli* by FrxA and HycI of *P. furiosus*. An *E. coli* cell-extract containing rSHI (PF0891-0894) as the only expressed *Pf* genes, was added to another cell-extract containing only either *FrxA* or *HycI* as the only expressed *Pf* genes. The mixtures were incubated for 30 min at the indicated temperature and the specific activity was determined at 80°C by the methyl viologen-linked assay.

To facilitate detection of heterologously-produced *Pf* SHI, all four vectors (Fig. 1b) were co-transformed into an *E. coli* strain MW1001 lacking the catalytic subunits of its own hydrogenases (*ΔhyaB ΔhybC ΔhycE*) [32]. Strain MW1001 produces no H_2_ during anaerobic growth, and cell extracts have no detectable hydrogenase activity at either 37 or 80°C (using the standard assay of methyl viologen (MV)-linked H_2_ evolution *vide infra*). However, cell-free extracts of anaerobically-grown *E. coli* MW1001 containing the four vectors encoding *Pf* SHI and the *Pf* hydrogenase processing genes did show hydrogenase activity at 80°C (Fig. 4a), which must therefore arise from recombinant *Pf* hydrogenase I.

**Figure 5:**
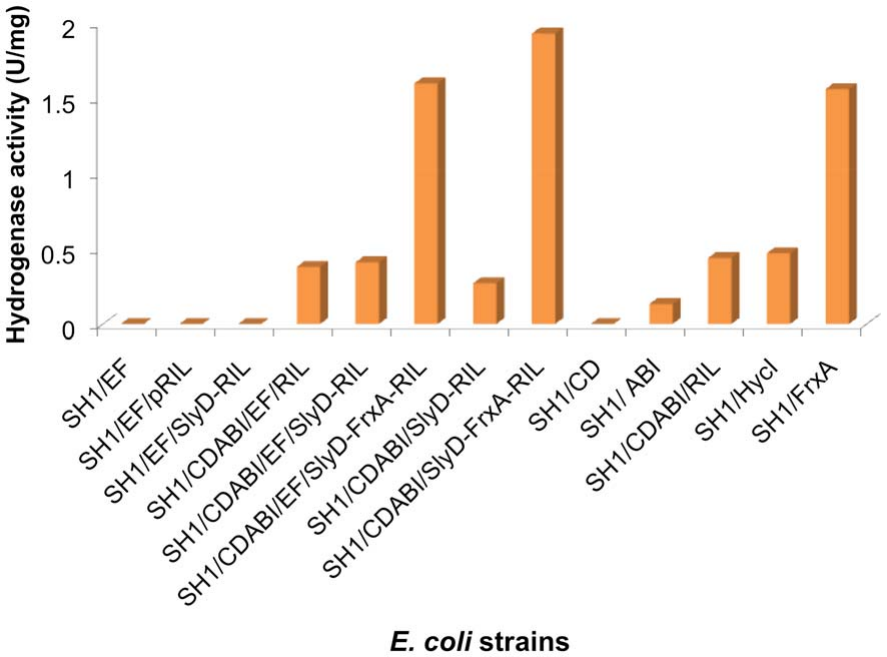
Analysis of the *P. furiosus* maturation proteins required to produce active rSHI in *E. coli*. The specific activities are shown for rSHI in cell extracts of *E. coli* resulting from the co-expression of different *Pf* processing genes in *E. coli* MW1001. Bars indicate MV-linked specific activity (µmol H_2_ evolved min^−1^ mg^−1^). All cell extracts were heated for 30 min at 80°C prior to assay.

In order to determine the minimum number of *Pf* genes needed for assembly of an active form of SHI in *E. coli*, different *Pf* accessory genes and plasmids were omitted from the complete heterologous expression system (Fig. 4a and Fig. 5). The results show that maximal recombinant production of functional SHI, as measured by the specific activity in the cell-free extract at 80°C, requires, in addition to the structural genes (PF0891-PF0894), coexpression of only the plasmid containing *frxA* (PF0975) (Fig. 4a). FrxA is, therefore, the protease required to process SHI by removing the four C-terminal –VVRL residues (Fig. 1a). Low activity was detected if HycI was present, but there was no activity if both proteases were absent, showing that *E. coli* proteases cannot process SHI. The *E. coli* processing enzymes HypABCD and HypEF appear to assemble a functional SHI whose catalytic subunit (PF0894) lacks C-terminal processing. This was confirmed both *in vivo* (Fig. 5) and using an *in vitro* assay (Fig. 4b) where extracts from *E. coli* cells expressing either only the four structural genes for SHI or only *frxA* were mixed and SHI activity was measured after incubation at 80°C. As expected, much lower SHI activity was obtained if *hycI* replaced *frxA*. Surprisingly, unprocessed SHI (where PF0894 lacks C-terminal cleavage) appears to be stable in *E. coli*, which will be of great utility to those interested in studying the mechanism of assembly of the [NiFe] site. The minimal expression system in *E. coli* therefore contains five *Pf* genes encoding only SHI and FrxA.

**Figure 6:**
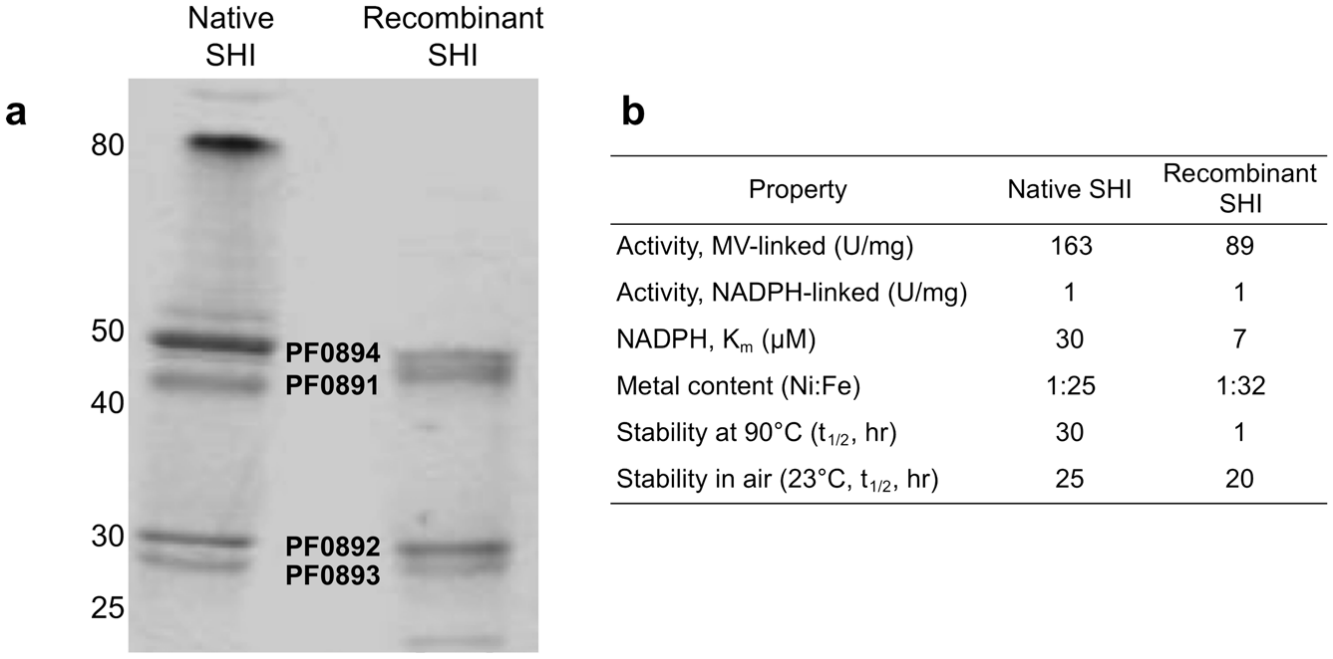
Properties of recombinant *P. furiosus* SHI. (**a**) Gel electrophoresis (SDS-PAGE) analysis of native and recombinant SHI. Bands representing the four subunits of the SHI are indicated, all of which were confirmed by MALDI-TOF analysis (data not shown). The high molecular weight band in the native protein, not seen in the recombinant version, represents undenatured tetrameric protein [11]. Recombinant PF0891 is approximately 2 kDa larger than the native protein due to the N-terminal extension (see Fig. 3). Molecular weight markers (kDa) are indicated. (**b**) Physical and catalytic properties of rSHI and the native hydrogenase purified from *P. furiosus* biomass. Hydrogen evolution was measured using either methyl viologen (MV) or NADPH as the electron donor at 80°C.

**Figure 7:**
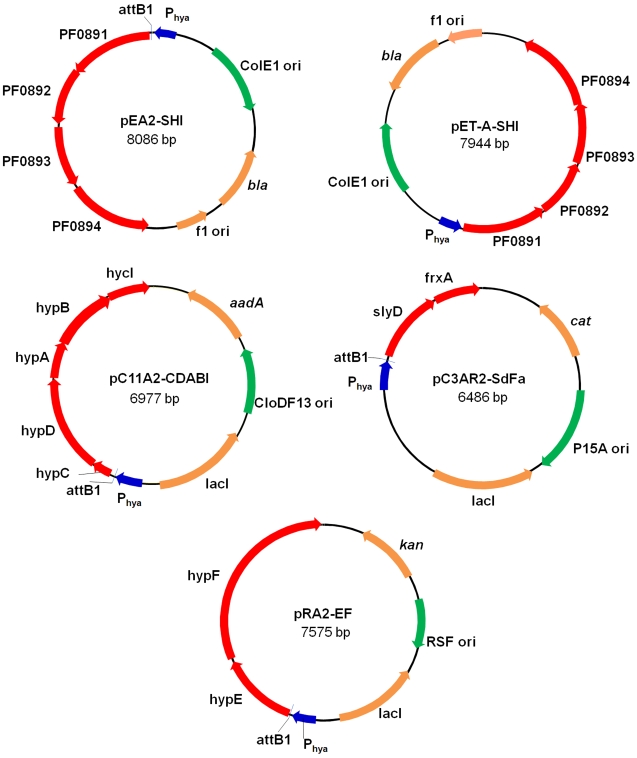
Maps of expression plasmids. Plasmid pEA2-SHI and pET-A-SHI were used to express rSHI in *E. coli*, and pET-A-SHI was used to generate SHI with a Gateway sequence and TEV recognition site attached to the N-terminal of PF0891 subunit. The plasmid pC11A2-CDABI contains the operons *hypCD* and *hypAB, hycI* from *P. furiosus*. The operons *hypCD* and *hypAB, hycI* of *P. furiosus* were linked by an intergenic region shown in Figure 3c. The Gateway sequence is located at the N-terminus of the HypC protein. The plasmid pC3AR2-SdFa has *slyD* and *frxA* of *P. furiosus* cloned behind P_hya_, the recombinant SlyD has the N-terminal Gateway sequence/TEV site. Plasmid pRA2-EF was constructed to co-express HypE/HypF, the same linker as in the plasmid pC11A2-CDABI or pC3AR2-SdFa, is inserted between *hypE* and *hypF* gene, and HypE contains the N-terminal Gateway sequence.

Recombinant *Pf*SHI (rSHI) was purified from *E. coli* strain MW4W (Fig. 6a, Table 2) to a final specific activity of 100 U mg^−1^, which is comparable to that of the native enzyme purified from *P. furiosus* biomass (Fig. 6b). The hydrogenase was obtained as a homogeneous protein of MW ∼150,000 Da (based on size-exclusion chromatography, data not shown), also similar to that of the native enzyme, and contained the four different protein subunits (Fig. 6a) [11]. Comparison of the properties of the recombinant and native enzymes (Fig. 6b) demonstrate that rSHI is similar in specific activity, affinity for NADPH (apparent K_m_), stability upon oxygen exposure, and metal content. The only significant difference is the lower stability of rSHI at 90°C. This was not due to the additional N-terminal residues (∼2 kDa) on one of the subunits (PF0891, due to the Gateway cloning strategy) since the properties of the wild-type form of rSHI, obtained by recloning PF0891-PF0894 to include only the wild-type amino acid sequence (pET-A-SHI, Fig. 7), and that of Gateway-tagged rSHI were virtually identical (data not shown).

**Table 2:** Purification of recombinant *P. furiosus* SHI from *E. coli*.

Step	Units (µmol min^−1^)	Protein (mg)	Specific Activity	Yield (%)	Purification (-fold)
			(Units mg^−1^)		
Cell Extract (CE)	1183	37260	0.03	100	1
Heat Treated CE	1287	2381	0.5	109	17
DEAE Sepharose	789	573	1	67	43
Phenyl Sepharose	237	45	5	20	166
Superdex S-200	80	0.8	100	7	3150

## Discussion

The ability of recombinant *Pf* SHI to accept electrons from the physiological electron carrier NADPH[22] is of some significance since this requires a properly processed, mature, and folded enzyme containing the FAD- (PF0892) and the FeS-containing (PF0891 and PF0892) subunits, in addition to catalytic LSU (PF0894) and SSU (PF0893). In contrast, electrons from the low potential artificial electron donor MV, which was used in all of the routine assays of hydrogenase activity, can theoretically be donated to any of the FeS clusters in the enzyme. The results (Fig. 6b) demonstrate that rSHI can accept electrons from NADPH as efficiently as the native enzyme, indicating that it is functionally folded and contains all cofactors.

Like other hydrogenases, *Pf* SHI is much more active with an artificial redox dye (methyl viologen) than with its physiological electron carrier ([34], see Figure 6), nevertheless, the specific activity of H_2_ production from NADPH by *Pf* SHI is more than 6-fold higher than that reported for an NADH-dependent hydrogenase [34]. While the recombinant form of SHI is as active as the native enzyme at 80°C, it is not as thermostable (Figure 6). Interestingly, the native enzyme gives rise after SDS-electrophoresis to a protein band of ∼80 kDa, in addition to the four subunits. This band retains hydrogenase activity and is thought to represent a highly stable SDS-resistant residual form of the holoenzyme ([11], and Lane 1 of Figure 6). The lack of this additional band in the recombinant preparations (Figure 6) suggests that assembly of the recombinant hydrogenase is not completely correct, even though its catalytic activity indicates that it is functionally assembled. It is possible that holoenzyme assembly to the native-like form of SHI represents a kinetic problem, as speculated with other recombinant hydrogenases [15], since all known *Pf* accessory genes are present in the recombinant host.

The availability of a system to produce rSHI provides a ready supply of the enzyme as well as the opportunity to generate mutant forms with desired characteristics. For example, it was recently demonstrated that a combination of NADPH-utilizing *Pf* SHI, obtained from native *Pf* biomass, with enzymes of the pentose phosphate pathway and starch phosphorylase, evolved H_2_ *in vitro* from starch with surprisingly high efficiency and yield [35]. Similarly, attempts have been made to link hydrogenase to the low potential reductant generated by photosynthesis, thereby constructing a light-driven H_2_-production system [36], [37]. A major problem is the generation of oxygen by photolysis of water, which inhibits the oxygen-sensitive hydrogenase. A functional expression system for hydrogenase provides the tools needed to engineer a hydrogenase enzyme resistant to O_2_ inactivation, as well for optimizing output of H_2_. Directed evolution techniques can now be utilized to generate [NiFe]-hydrogenases with tailored catalytic activity, oxygen sensitivity, and potentially even changing coenzyme specificity. There are also broader implications for heterologous production of complex, heteromeric metalloproteins. If *E. coli* does not possesses homologs of the required accessory genes for any candidate metalloprotein complex, coexpression of multiple genes involved in multiprotein complex assembly is now possible even under anaerobic conditions, and even for complex metalloproteins from extremophiles. While expression of stable hyperthermophilic proteins in *E. coli* is well-documented (e.g., see [38]), it is surprising that at least some of them, such as the *Pf* protease, are functional at 37°C, in this case to efficiently process *Pf* SHI in *E. coli*. The ability to heterologously produce and engineer a soluble hydrogenase, particularly one as thermostable and as relatively oxygen-insensitive as *Pf* SHI, makes this enzyme an excellent model system for future basic and also applied studies (e.g., [39]).

## Methods

### Strains and growth conditions

*Escherichia coli* strain MW1001 (*lacI* ^q^ *rrnB*_T14_ *ΔlacZ*_WJ16_ *hsdR514 ΔaraBAD*_AH33_ *ΔrhaBAD*_LD78_ *ΔhyaBΔhybCΔhycE*) [32] was a generous gift from Dr. Thomas K. Wood (Department of Civil and Environmental Engineering, Texas A & M University). *E. coli* strains were routinely grown in 2X YT [40] medium containing Ampicillin 50 µg/mL, Chloramphenicol 20 µg/mL, Streptomycin 25 µg/mL, Kanamycin 25 µg/mL, supplemented with 1 mM MgSO_4_. For anaerobic expression, an overnight culture was used to inoculate (1% vol/vol) 1 L of medium in a 2.8 L baffled Fernbach flask, and the culture was grown at 37°C shaking at 150 rpm under N_2_ for 3–5 hours to an OD_600nm_ ∼0.8. Cultures were then decanted into 1 L bottles and 100 µM FeCl_3_, 25 µM NiSO_4_ and 56 mM glucose were added. The bottles were sealed with 43 mm Straight Plug stoppers (Wheaton, Millville, NJ) under N_2_, and incubated at 37°C with shaking at 120 rpm for 12–16 hours. For larger scale fermentations (15 L), cultures were grown aerobically as described above and were decanted into a carboy (20 L) and sealed under N_2_. The culture was allowed to grow anaerobically to induce expression and maturation of the recombinant hydrogenase for 14 hours. Cells were harvested by centrifugation at 10,000×*g* for 5 min. The cell pellets were flash frozen in liquid nitrogen and stored at −80°C before use. Molecular biology techniques were performed as described [40], and unless otherwise indicated, chemicals were obtained from Sigma-Aldrich (St. Louis, MO). All molecular biology reagents were utilized following the manufacturer's protocols and were obtained from New England Biolabs (Ipswich, MA), except for Pfu DNA polymerase (Stratagene, La Jolla, CA) and the reagents for GATEWAY™ recombination (Invitrogen, Carlsbad, CA).

### Purification of recombinant *P. furiosus* SHI

All purification steps were performed under strictly anaerobic conditions. Frozen cell pellets were resuspended in Buffer A (50 mM Tris, 2 mM sodium dithionite (J.T. Baker, Phillipsburg, NJ), pH 8.0) containing 0.5 mg/mL lysozyme (Sigma) and 50 µg/mL deoxyribonuclease I (Sigma) in a ratio of 3 mL/g wet weight cells. The resuspended cells were incubated with stirring at room temperature under an atmosphere of argon for one hour and sonicated on ice with a Branson Sonifer 450 for 30 minutes (output 6.0, 50% duty cycle). Cell lysates were heated to 80°C for 30 minutes, cooled to room temperature, and centrifuged (Beckman L90K ultra centrifuge 45Ti rotor at 100,000×*g* for 1 hr). The supernatant was collected and loaded onto a DEAE anion exchange column (66 mL; GE Healthcare, Piscataway, NJ) equilibrated in Buffer A. The column was washed with 5 volumes of Buffer A and the proteins were eluted with a gradient of 0 to 500 mM NaCl in Buffer A over 20 column volumes and collected in 25 ml fractions in an anaerobic chamber. Fractions containing hydrogenase activity were pooled and Buffer A containing 2.0 M ammonium sulfate was added to a final concentration of 0.8 M. The sample was then loaded on to a column of Phenyl Sepharose (45 ml; GE Healthcare) equilibrated in Buffer C (Buffer A containing 0.8M (NH_4_)_2_S0_4_). The column was washed with 5 volumes of Buffer C and eluted with a 20 column volume gradient from 0.8-0 M (NH_4_)_2_SO_4_ in Buffer A collected in 20 ml fractions. Fractions containing hydrogenase activity were combined and concentrated anaerobically by ultrafiltration (PM-30 kDa MWCO membrane, Millipore, Billerica, MA), and applied to a gel filtration column (Superdex S-200 26/60 GE Healthcare) equilibrated with Buffer D (Buffer A containing 300 mM NaCl) and eluted anaerobically in 2 mL fractions over 1.5 column volumes. Native *P. furiosus* SHI was isolated as previously described [41]. The results of the procedure are summarized in Table 2.

### Hydrogenase assays

Hydrogen production by *E. coli* cultures was routinely measured by removing 100 µl from the culture headspace with a gas-tight syringe (Hamilton Co, Reno, NV) and injecting into a Shimadzu GC8 gas chromatograph equipped with a washed molesieve 5A 80/100 (6′×1/8″×0.085″) column. Hydrogenase activity was routinely determined by H_2_ evolution from methyl viologen (1 mM) reduced by sodium dithionite (10 mM) at 80°C as described previously [41], except the buffer was 100 mM EPPS, pH 8.4. For the physiologically-relevant hydrogen evolution assay, methyl viologen and sodium dithionite were replaced by NADPH (1 mM). One unit of hydrogenase specific activity is defined as 1 µmole of H_2_ evolved min^−1^ mg^−1^. Oxygen sensitivity assays were performed by exposing samples to air at 25°C. Thermal stability assays were measured by anaerobic incubation of the hydrogenase samples (0.06 mg/ml in 100 mM EPPS buffer, pH 8.4, containing 2 mM sodium dithionite) at 90°C. Residual enzyme activities were measured using the MV-linked H_2_-evolution assay.

### Construction of lacZ reporter anaerobic expression plasmids

Four *E. coli* anaerobic promoters, P_hya_, P_hyb_, P_hyc_ and P_hyp_ (Fig. 2) were inserted into the plasmid pLX1 between *Eco*RI and *Xma*I sites to construct plasmids pLXA, pLXB, pLXC and pLXP. pLX1 was constructed from plasmid pSB2019 [33] by replacing a green fluorescent protein gene between *Xma*I and *Sal*I with the N-terminal sequence of lacZ. Plasmids containing lacZ under control of these promoters were transformed into *E. coli* DH10B cells. β-galactosidase activity in extracts of anaerobically-grown cells was analyzed (Spectra MAX 190 96 well microplate reader) using a modified Miller assay [30]. The specific activity of β-galactosidase is expressed as units per mg of total protein in the cell-free extract.

### Construction of anaerobic expression vectors

A series of four vectors were constructed by Horanyi et al. [25] in which the GATEWAY™ recombination cassette from Invitrogen was combined with the four compatible expression vectors of the Novagen (EMD Chemicals, San Diego, CA) DUET vector system (Table 1). The four original DUET vectors, as well as the four GATEWAY-modified versions (pDEST-C3A, pDEST-C11A, pRSF-ACG, and pET-ACG), were further modified to replace the native T7 promoter, which is not functional under anaerobic growth conditions [42]. The *hya* promoter [43] (see Fig. 3) was amplified from genomic DNA purified from the *E. coli* strain MG1655 using the primers (JS1 5′-CTCGAATTCCTTCTCTTTTACTCGTTTAG, JS2 5′-CACCCATATCGCACGTCTCTCCTCC). The PCR product was then treated with T4 polynucleotide kinase, and ligated to plasmid pDEST-C3 digested with *Eco*ICRI to create pDEST-C3A; the entire cassette containing P_hya_ and Gateway cloning sites was amplified with primers (JS1 and JS3 5′-GCCGCAAGCTTAGCAGCCGGAT, the cassette was digested with *Eco*RI and *Hind*III, then ligated with the similarly digested plasmids (pCDFDuet1, pRSFDuet1 and pET23) to create pDEST-C11A, pRSF-ACG and pET-ACG respectively. A 813 bp DNA fragment containing three tRNA genes from plasmid pRIL [44] was amplified using the primers JS4 (5′-CGCGGATCCGTCACCCTGGATGCTGTAC) and JS5 (5′-AAGTACGAGCTCAGGTCGCAGACGTTTTGCA), and cloned into pDEST-C3A between *Bam*HI and *Sac*I to create the plasmid pC3A-RIL (Table 1).

### Cloning of the *P. furiosus* genes encoding SHI and the hydrogenase maturation proteins

The *P. furiosus* SHI structural genes (PF0891-PF0894) were amplified using purified *P. furiosus* genomic DNA (DSM 3638, Genbank accession number AE009950) as the template. The entire four-gene operon was amplified with the primers (PF0891-attB1-TEV: 5′-GGGGACAAGTTTGTACAAAAAAGCAGGCTCAGAAAACCTGTATTTTCAGGGAGGAAGGTATGTTAAGTTACCCAAGG and PF0894-attB2-TEV: 5′-GGGGACCACTTTGTACAAGAAAGCTGGGTCTCCTCCCTGAAAATACAGGTTTTCCTAAAGTCTAACCACGTGGACTGAG). The 4164 bp PCR product was crossed into the GATEWAY entry vector pDONR/Zeo using the manufacturer's protocol, resulting in the entry vector pDONR-SHI, and subsequently used to cross into the modified GATEWAY expression vector pET-SHI. The plasmid pET-A-SHI was constructed to produce “tagless” recombinant *P. furiosus* SHI. P_hya_ without the downstream GATEWAY tag was amplify with primer pair Pa-SacF (CTCGAATTCCTTCTCTTTTACTCGTTTAG) and Pa-HindR (TGAGGAAGCTTATCGATGGTACCGCGGCATGCATATGGCACGTCTCTCCTCCTTGCG), the pET-A plasmid was made by inserting the *Hind*III/*Eco*RI digested PCR product into the similarly treated pETDuet-1(Novagen). The tagless Pf SHI gene was amplified with primer pair 0891-NdeF (GATAGGTTCCATATGAGGTATGTTAAGTTACCCAAGGA) and 0894-KpnR (ATAGGGTACCTTAAAGTCTAACCACGTGGACTGAGC), the *Nde*I/*Kpn*I digested PCR product was inserted into similarly treated pET-A to produce pET-A-SHI. To make the expression vector pC11A-CDABI, the *Pf hypCD* operon (PF0548-PF0549) was first amplified with primers PF0548-attB1-TEV (5′- GGGGACAAGTTTGTACAAAAAAGCAGGCTCAGAAAACCTGTATTTTCAGGGAGGATGCCTTGCAATCCCAGGGAAAG) and CD-ABI-R (5′-CTTACTATTGCATCTGCCAACGCCCATTCGTGCATTTTCCACCTCCTACATCAGGGCGCCATATTTGTAAA). This 1460 bp *hypCD* PCR product was used as the forward primer to join and amplify the *hypABI* operon (PF0615 – PF0617) creating the artificial *hypCDABI* operon with the reverse primer PF0617-attB2-TEV (5′- GGGGACCACTTTGTACAAGAAAGCTGGGTCTCCTCCCTGAAAATACAGGTTTTCCTAAGAAAGCTCAAGACTTTCATAA). A fused *hypEF* operon (PF0604 and PF0559) was obtained with the same strategy by PCR that employed the primer PF0604-attB1-TEV (5′-GGGGACAAGTTTGTACAAAAAAGCAGGCTCAGAAAACCTGTATTTTCAGGGAGGAGAAGAACTAATTAGGGAGGTAA and the primer EF-fusion (5′-GTGAATTCTATAAGCTTTCATTCTCTCCCCCAGATACATTTTCCACCTCCTAACAAACTCTAGGAACGGGATCAC). The resulting 1025 bp PCR product of PF0604 was used as the forward primer along with the reverse primer PF0559-attB2-TEV (5′-GGGGACCACTTTGTACAAGAAAGCTGGGTCTCCTCCCTGAAAATACAGGTTTTCCTACATCAGGGCGCCATATTTGTAA) to generate the 3428 bp artificial *hypEF* operon. The fused *P.furiosus slyD* (PF1401) and *frxA* (PF0975) genes were cloned by the same PCR method with the primers PF1401-attB1-TEV (5′-GGGGACAAGTTTGTACAAAAAAGCAGGCTCAGAAAACCTGTATTTTCAGGGAGGAAAAGTAGAGAAAGGAGATGTCA), the fusion primer SlyD-FrxA-F (5′-GAGGAGAGTGAGTCTAAAGCGGAAGAATCTTAAGAGGTGGAAAGTGAGTAACTTTTTAAACTTTCACTT) and the reverse primer frx-attB2 (5′-GGGGACCACTTTGTACAAGAAAGCTGGGTCTCCCTATTACTCA). It was not possible to transform all four plasmids simultaneously into *E. coli* strains, so routinely one plasmid was transformed, and competent cells made from these cells to transform the next plasmid, and this process repeated as needed.

### Metal and Proteomic Analyses

Nickel and iron were measured using a quadrupole-based ICP-MS (7500ce, Agilent Technologies, Tokyo, Japan), equipped with a MicroMist Nebulizer (Agilent Technologies). Proteins were separated using native- or SDS-PAGE gradient gel electrophoresis (4–20% Criterion gels; Biorad, Hercules, CA) and stained with Coomassie Brilliant Blue. Gel bands of interest were cut out, processed and digested for 16 h at 37°C according to the manufacturer's protocol provided with the recombinant porcine trypsin used for the in-gel protein digest (Roche Applied Science, Indianapolis, IN). The peptides were purified with C-18 reversed-phase NuTip® cartridges according to the manufacturer's instructions (Glygen Corp., Columbia, MD). Approximately 1 µL of purified peptides and 1 µL of ProteoMass Peptide & Protein MALDI-MS Calibration Kit standard (Sigma) were spotted onto a MTP 384 Massive MALDI target (Bruker Daltonics, Billerica, MA). The target was analyzed using a Bruker Daultonics Autoflex MALDI-TOF mass spectrometer (Billerica, MA) following calibration with the standard. The mass list was generated by the SNAP peak detection algorithm using a signal-to-noise threshold of four following baseline correction of the spectra. Proteins were identified by searching the mass list against the National Center for Biotechnology Information (NCBI) annotation of the *P. furiosus* genome (NC_003413) using Mascot's Peptide Mass Fingerprint tool (version 2.1, Matrix Science Ltd., Boston, MA). The searches were conducted using a peptide mass tolerance of 1.0, variable modifications of Carbamidomethylation (C) and Oxidation (M), and a maximum of one missed cleavage. Proteins with a p<0.05 (corresponding to a Mascot protein score of greater than 46) were considered significant.
